# aThe dyslexia candidate gene DYX1C1 is a potential marker of poor survival in breast cancer

**DOI:** 10.1186/1471-2407-12-79

**Published:** 2012-02-29

**Authors:** Gustaf Rosin, Ulf Hannelius, Linda Lindström, Per Hall, Jonas Bergh, Johan Hartman, Juha Kere

**Affiliations:** 1 Department of Biosciences and Nutrition, Novum, and Science for Life Laboratory, Karolinska Institutet, Hälsovägen 7, 141 83 Huddinge, Sweden; 2 Department of Medical Epidemiology and Biostatistics, Karolinska Institutet, Box 281, 17177 Stockholm, Sweden; 3 Department of Oncology-Pathology, Radiumhemmet, Karolinska Institutet, Karolinska University Hospital, 17176 Stockholm, Sweden; 4 Department of Medical Genetics, University of Helsinki, and Folkhälsan Institute of Genetics, Helsinki, Finland; 5 Mutation Analysis Core Facility, Clinical Research Centre, Karolinska Institutet, 141 83 Huddinge, Sweden

**Keywords:** DYX1C1, Breast cancer, Estrogen receptor, Dyslexia

## Abstract

**Background:**

The dyslexia candidate gene, *DYX1C1*, shown to regulate and interact with estrogen receptors and involved in the regulation of neuronal migration, has recently been proposed as a putative cancer biomarker. This study was undertaken to assess the prognostic value and therapy-predictive potential of DYX1C1 mRNA and protein expression in breast cancer.

**Methods:**

DYX1C1 mRNA expression was assessed at the mRNA level in three independent population-derived patient cohorts. An association to estrogen/progesterone receptor status, Elston grade, gene expression subtype and lymph node status was analyzed within these cohorts. DYX1C1 protein expression was examined using immunohistochemistry in cancer and normal breast tissue. The statistical analyses were performed using the non-parametric Wilcoxon rank-sum test, ANOVA, Fisher's exact test and a multivariate proportional hazard (Cox) model.

**Results:**

DYX1C1 mRNA is significantly more highly expressed in tumors that have been classified as estrogen receptor α and progesterone receptor-positive. The expression of DYX1C1 among the molecular subtypes shows the lowest median expression within the basal type tumors, which are considered to have the worst prognosis. The expression of DYX1C1 is significantly lower in tumors graded as Elston grade 3 compared with grades 1 and 2. DYX1C1 protein is expressed in 88% of tumors and in all 10 normal breast tissues examined. Positive protein expression was significantly correlated to overall survival (Hazard ratio 3.44 [CI 1.84-6.42]) of the patients but not to any of the variables linked with mRNA expression.

**Conclusion:**

We show that the expression of DYX1C1 in breast cancer is associated with several clinicopathological parameters and that loss of DYX1C1 correlates with a more aggressive disease, in turn indicating that DYX1C1 is a potential prognostic biomarker in breast cancer.

## Background

Breast cancer is a heterogeneous disease consisting of several distinct subtypes with characteristic gene expression patterns resulting in differences in overall survival [1,2].

Several clinicopathological variables are routinely examined with a breast cancer diagnosis, including estrogen receptor (ER), progesterone receptor (PR), HER2/neu amplification, lymph node involvement and histopathological grade [3]. Discussions about the inclusion of proliferation markers such as Ki67 and Cyclin A are ongoing [3,4] One of the most important clinical parameters is ERα. This is both a prognosticator and a therapy predictor; the majority of breast cancers are considered ERα-positive at time of diagnosis. ERα-positive tumors benefit from endocrine therapy and correlate with increased survival [5]. However, many women either fail to respond or develop resistance to the endocrine therapy. Early prediction of endocrine sensitivity is important for the selection of adjuvant therapy, or decision to use additional systemic adjuvant therapy [6].

Recently Kim *et al. *proposed *dyslexia susceptibility 1 candidate 1 *(*DYX1C1) *as a potential cancer biomarker after comparing splice variant-specific RNA levels in a variety of different human tumors and normal samples [7]. Shortly afterwards, Chen *et al. *demonstrated that DYX1C1 was increasingly expressed in malignant breast tumors compared with benign tumors. However, due to the low power of the statistical analysis, the authors were unable to look for differences between different sub-categories of tumors or into clinicopathological features [8].

In 2003, *DYX1C1 *was described as the first candidate gene for developmental dyslexia by Taipale *et al. *[9]. *DYX1C1 *has a length of 78 kb and consists of 10 exons which encode a protein of 420 amino acids. The protein contains three TPR motifs and one p23 domain, all commonly known for their protein-protein interactions [10], but overall, DYX1C1 does not belong to any structurally defined gene family. In cell lines, DYX1C1 has been shown to interact with the intracellular chaperones Hsp70, Hsp90, and the ubiquination ligase CHIP [10,11]. The interaction with Hsp70 and Hsp90 has also later been shown in malignant breast tumors [8]. When overexpressed in the SH-SY5Y line, DYX1C1 interacts with and regulates both the levels of ERα as well as the second estrogen receptor, *ESR2 *(ERβ) in a dose-dependent fashion, likely through an interaction with CHIP [12].

Many of the genes associated with dyslexia have been linked to neuronal migration and axonal guidance. By using *in utero *siRNA, knocking down *DYX1C1 *in the developing mice brain, the migration of neurons was halted [13]. Furthermore, data from our group indicate that when DYX1C1 is overexpressed in the neuroblastoma cell line SH-SY5Y, the motility of the cells increased (unpublished data).

Kim *et al. *[7] and Chen *et al. *[8] introduced DYX1C1 as a possible breast cancer biomarker. In this study, our aim has been to examine the expression of both DYX1C1 mRNA and protein in breast cancer tumors and normal mammary tissue and analyze their correlation to several predictive and prognostic markers.

## Methods

### Patients

#### CHARES

The samples used for this study represented a subset of 61 randomly selected patients who were included in a population-based case-control study where all women diagnosed with invasive breast cancer between the ages of 50 and 74 years living in Sweden between October 1st 1993 and May 31st 1995 were asked to participate [14]. Of our 61 samples, 47 were classified as ER-positive (77%) and 14 as ER-negative (23%). Other analyzed clinical variables are summarized in Table 1. The ethical committee of the Karolinska Institutet, Stockholm, Sweden, has approved gene expression analysis of this cohort.

**Table 1:** Patient collections and available clinicopathological data.

Parameter	CAHRES (n = 61)	Uppsala (n = 315)	Stockholm (n = 159)
qRT-PCR (DYX1C1)	+	-	-
Affymetrix microarray	-	+	+
IHC	-	+	-
ERα-status	+	+	+
PR-status	+	+	-
Lymph node status	+	+	-
Periglandular status	+	-	-
Subtype analysis	-	+	+
Elston grade	+	+	+
Survival data	-	+	-

Summarizes the data available for analysis among the three different cancer collections used in this study. + = data available, - = data not available, qRT-PCR = quantitative reverse transcriptase polymerase chain reaction, IHC = Immunohistochemistry, ERα = Estrogen receptor alpha, PR = Progesterone receptor

#### Uppsala breast cancer patient cohort

Primary tumor samples from 315 women were collected representing 65% of surgically resected breast cancers in the county of Uppsala, Sweden, between January 1st 1987 and December 31st 1989. Of these, 253 women had a sufficient quantity of RNA to perform microarray gene expression profiles and passed quality control [2].

Based on gene expression, the tumors were classified into the subtypes proposed by Sørlie *et al. *[1]. The microarray data has previously been deposited at the Gene Expression Omnibus (GEO), with accession code GSE3494.

Clinicopathological parameters were obtained from patient records or routine diagnostic measurements, including several morphological parameters and histopathological grade. The population-derived patient cohort has been more thoroughly described elsewhere [15]. The ethical committee of the Karolinska Institutet, Stockholm, Sweden, has approved the microarray profiling.

#### Stockholm breast cancer patient cohort

524 women with breast cancer were included in the study sample, which represents all patients operated at Karolinska Hospital between January 1st 1994 and December 31st 1996. By taking into consideration several exclusion criteria, expression profiles from 159 tumors were finally available [16]. The microarray data has previously been deposited at Gene Expression Omnibus (GEO) with accession code GSE1456. Clinicopathological measurements were obtained from the Stockholm-Gotland cancer registry and from patient records (summarized in Table 1). The population-derived patient cohort has been more thoroughly described elsewhere [16]. The ethical committee at Karolinska Hospital, Stockholm, Sweden, has approved the microarray profiling.

### Real-Time qRT-PCR

#### RNA extraction, cDNA synthesis and real-time PCR

RNA was extracted from snap frozen tumors using RNeasy fibrous mini kit (Qiagen, Hilden, Germany), with minor modifications from the manufacturer's instructions. The integrity and concentration of the RNA was analyzed with the Agilent 2100 Bioanalyzer (Agilent Technologies, Rockville, MD, USA) and stored at -70°C. All 61 samples had a RNA integrity number (RIN) > 8 and a 28S/18S ratio ≥ 1.7, indicating that the RNA quality was high. cDNA was synthesized using SuperScript™ III Reverse Transcriptase reagents (Invitrogen, Carlsbad, CA, USA), according to the manufacturer's instructions. Input RNA from each tumor did not exceed the maximum allowed amount, final reaction volume was 20 μL. Incubation conditions were as follows; 25°C for 10 minutes and 42°C for 60 minutes. Reaction was terminated by incubating the samples at 85°C for 5 minutes, diluted with water to 100 μL and stored at -20°C.

The quantitative reverse transcriptase PCR (qRT-PCR) was performed in triplicates on the 7500 Fast Real-Time PCR system using TaqMan probes according to standard protocols (Applied Biosystems, Carlsbad, CA, USA). DYX1C1 expression was examined by TaqMan assay (Hs00370049_m1) and GAPDH TaqMan assay (Hs99999905_m1) was used for normalization (Applied Biosystems). All other materials and buffers were acquired from Applied Biosystems. The thermal cycling condition were 95°C for 20 seconds once, then repetitively 95°C for 3 seconds and 60°C for 30 seconds. Final sample volume was 10 μL, the method have been described more thoroughly elsewhere [17].

### Affymetrix gene expression array

Gene expression profiling was performed using Affymetrix HG-U133 A and B arrays (Affymetrix, Santa Clara, CA, USA) according to the manufacturer's instructions. The procedure has been described elsewhere [16,18]. Raw data was normalized using the MAS5 global mean method, expression values were calculated for each gene and transformed using the natural log. Annotation of probes was performed by using HG-U133A/B release 30 (Affymetrix). The Probe "235273_at" that detects the 3 RefSeq isoforms of DYX1C1 and the probe "205225_at" that detects the 4 RefSeq isoforms of *ESR1 *(ERα) were used to analyze the expression of these genes.

### Immunohistochemistry

Tissue microarrays (TMAs) constructed from the Uppsala breast cancer patient cohort were used for immunohistochemical (IHC) staining. Two sections from each patient from a representative part of the tumor were collected to construct the TMAs.

The formalin fixed paraffin-embedded TMAs were de-paraffinized using xylene and rehydrated in an ethanol gradient. Epitopes were retrieved in heated citric buffer (pH 6.0) for 20 min. Slides were blocked with 1% BSA for 1 h and 0.5% H_2_O_2 _for 30 minutes and exposed to the rabbit anti-DYX1C1 antibody (Proteintech, Manchester, UK) over night at 4°C. Biotinylated anti-rabbit antibody was used as the secondary antibody (Sigma-Aldrich, St. Louis, MO, USA). The avidin-biotin complex method was used to amplify the signal (Vector Laboratories, Burlingame, CA, USA) and together with 3,3'-Diaminobenzidine (DAB) (Dako, Glostrup, Denmark) to detect the DYX1C1/antibody interaction in the tissue sections. The slides were finally counterstained with hematoxylin.

The staining was analyzed independently by two researchers using the Allred scoring method [19,20]. Sections were given a proportion score between 0 and 5 representing the estimated relative number of positive tumor cells: none = 0, < 1/100 = 1, 1/100 to 1/10 = 2, 1/10 to 1/3 = 3, 1/3 to 2/3 = 4, > 2/3 = 5. An intensity score was also given: none = 0, weak = 1, moderate = 2, strong = 3, representing the relative intensity of the positive tumor cells. The scores were added together and the mean score of the two observations was used to obtain a final total score, ranging between 0 and 8. If the two observers disagreed with the scoring, the sections were reexamined until both observers concurred.

### Statistical analysis

DYX1C1 mRNA expression was calculated using the ΔCt method by subtracting the mean CT-value of triplicates of the normalizing gene (GAPDH) from the mean of triplicates of DYX1C1. 2^-ΔΔCT ^(fold change) was calculated by using ΔCt.

The non-parametric Wilcoxon rank-sum test for independent samples was used when comparing a continuous variable between two different categorical clinicopathological characteristics such as ERα, PR, lymph node, and Elston grade. This test was performed instead of a Student's T-test to better withstand the effects of non-normal distribution in the data, however, the robustness of the test normally results in higher P-values compared with Student's T-test. The ANOVA method was used when comparing a continuous outcome variable with several categorical explanatory variables. A multivariate linear regression model was also fitted with an mRNA expression of DYX1C1 as the dependent variable.

Differences between specific patient groups regarding clinicopathological characteristics such as lymph node metastasis, ERα, progesterone receptor (PR), and P53 mutation in relation to DYX1C1 protein expression (from IHC analyses) were determined using Fisher's exact test. Patients were categorized into positive by a value of an Allred score > = 1 or negative by an Allred score < 1.

The Kaplan-Meier method was used for univariate analyses of overall survival, from primary breast cancer diagnosis until death (including both breast cancer-specific death and other), or until the end of follow-up of the study (November 1st, 1999). Patients were stratified according to positive or negative DYX1C1 protein expression. The risk of dying in relation to DYX1C1 protein expression was modeled by use of a multivariable proportional hazard (Cox) model, adjusting for available potential confounding factors on survival, such as age, ERα status, PR status, Elston grade, and lymph node metastasis. The proportional hazard assumption for the main exposure variable was assessed using Schoenfeld's test statistics [21]. No statistically significant deviation was noted for the main exposure as studied.

An arbitrary level of 5% for statistical significance (two-tailed) was used in all analyses. R (2.10.1) [22], or SAS (9.2) software was used for statistical calculations.

## Results

### Correlations of DYX1C1 mRNA levels and clinicopathological parameters

#### Estrogen receptor status

**Figure 1:**
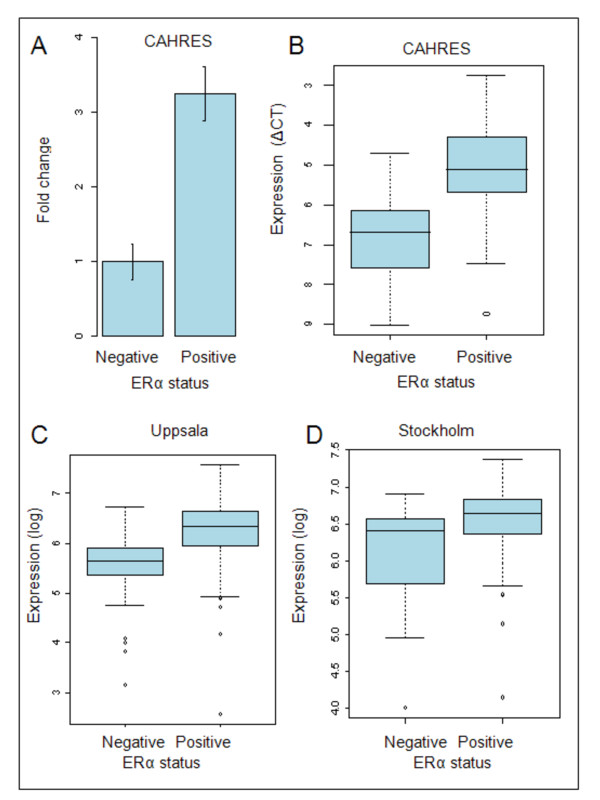
**Distribution of DYX1C1 mRNA expression stratified by ERα status**. (A) Fold change of DYX1C1 mRNA when comparing the expression between ERα-positive and -negative tumors in CHARES. GAPDH was used for normalization. The bars indicate 1 standard deviation. (B) Boxplot of the distribution of DYX1C1 mRNA expression examined by qRT-PCR in the CAHRES cohort between ERα-positive and ERα-negative tumors. The expression of DYX1C1 is significantly higher in ERα-positive tumors (note that the y-axis is inverted in CAHRES, were a lower dCT value indicates a higher expression). (C) Boxplot of the distribution of DYX1C1 mRNA expression examined by Affymetrix microarray in the Uppsala cohort between ERα-positive and ERα-negative tumors. The expression is significantly higher in ERα-positive tumors. (D) Boxplot of the distribution of DYX1C1 mRNA expression examined by Affymetrix microarray in the Stockholm cohort between ERα-positive and ERα-negative tumors. The expression is significantly higher in ERα-positive tumors.

In order to quantify DYX1C1 mRNA expression we performed qRT-PCR of the 61 breast cancer tumors in the CAHRES patient cohort and calculated the ΔC_T. _When comparing the ΔC_T _values, the mean DYX1C1 mRNA expression was significantly correlated to ERα status (p < 0.0001). The expression of DYX1C1 in ERα-positive tumors was 3.2 times higher compared with ERα-negative tumors (Figure 1).

Furthermore, we examined the differences in DYX1C1 expression in two independent breast cancer patient cohorts, from Uppsala and Stockholm, where transcriptome gene expression microarrays had been performed. Similar to our qRT-PCR results, DYX1C1 expression was found to be significantly higher in the tumors biochemically classified as ERα-positive tumors compared with ERα-negative in both of the cohorts (Figure 1). In the Uppsala breast cancer patient cohort, the mean DYX1C1 expression was 68% higher in ERα-positive tumors compared with ERα-negative (p < 0.001). Similarly, in the Stockholm Cohort, the expression was on average 61% higher in ERα-positive tumors compared with ERα-negative tumors (p < 0.05).

#### Progesterone receptor status

**Figure 2:**
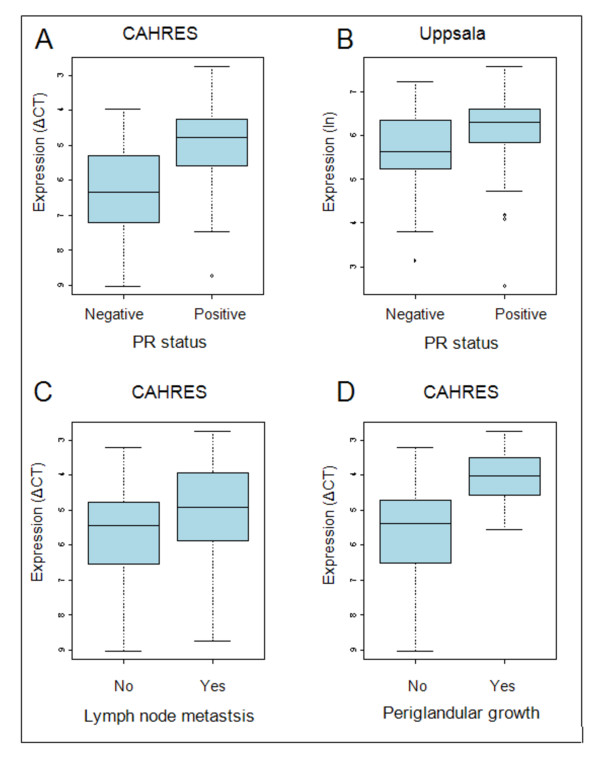
**Distribution of DYX1C1 mRNA expression stratified by progesterone receptor status and lymph node involvement**. (A) Boxplot of the distribution of DYX1C1 mRNA expression examined by qRT-PCR in the CAHRES cohort between PR-positive and PR-negative tumors. The expression of DYX1C1 is significantly higher in PR-positive tumors. Note that the y-axis is inverted, a lower dCT value indicates higher expression. (B) Boxplot of the distribution of DYX1C1 mRNA expression examined by microarray in the Uppsala cohort between PR-positive and PR-negative tumors. The expression of DYX1C1 is significantly higher in PR-positive tumors. (C) Boxplot of the distribution of DYX1C1 mRNA expression examined by qRT-PCR in the CAHRES cohort between patients with positive lymph node metastasis and negative lymph node metastasis. The expression of DYX1C1 is significantly higher in tumors of lymph node-positive patients. Note that the y-axis is inverted, a lower dCT value indicates higher expression. (D) Boxplot of the distribution of DYX1C1 mRNA expression examined by qRT-PCR in the CAHRES cohort between patients with positive and negative periglandular growth. The expression of DYX1C1 is significantly higher in patients with periglandular growth. Note that the y-axis is inverted, a lower dCT value indicates higher expression.

In the CAHRES patient cohort, the expression of DYX1C1 mRNA was significantly higher in tumors classified as PR-positive (p < 0.001). The expression of DYX1C1 was on average 2.6 times higher in PR expressing tumors compared with non-expressing tumors (Figure 2).

Similar results were seen in patients in the Uppsala patient cohort when stratified according to PR status. The expression of DYX1C1 was 48% higher in PR-positive tumors (p < 0.05) compared with PR-negative tumors (Figure 2). We were unable to correlate DYX1C1 expression to the PR status in the Stockholm patient cohort as no PR data was available.

#### Lymph node status

In the CAHRES patient cohort, DYX1C1 mRNA expression was significantly higher in patients with at least one lymph node metastasis (p < 0.05) (Figure 2). Additionally, DYX1C1 mRNA was more highly expressed in patients with tumors showing periglandular growth of one or more lymph nodes (p < 0.01) (Figure 2). This indicates that metastatic cells had begun penetrating the lymph node capsule.

In the Uppsala cohort, 84 patients were node-positive and 160 node-negative. No significant correlation with DYX1C1 mRNA expression and lymph node metastasis was observed in this patient cohort similar to the qRT-PCR data from the CAHRES patient material (data not shown). No data on lymph node status were available for the Stockholm patient cohort, data on periglandular growth for the Uppsala or Stockholm patient cohorts were also not available. These correlations could, therefore, not be examined within these data sets.

#### DYX1C1 expression in breast cancer subtypes

**Figure 3:**
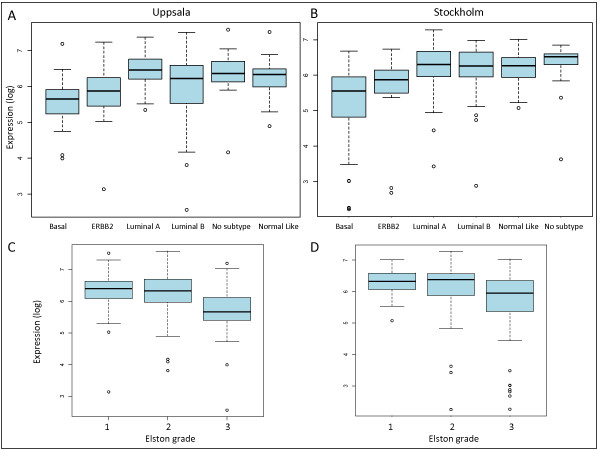
**Analysis of DYX1C1 mRNA expression among tumor subtype and Elston grade**. (A) Boxplot of the distribution of DYX1C1 mRNA expression examined by Affymetrix microarray in the Uppsala cohort between the different breast cancer subtypes. Lowest median expression is seen in the basal subtype, whereas the highest is seen in the luminal A subtype. (B) Boxplot of the distribution of DYX1C1 mRNA expression examined by Affymetrix microarray in the Stockholm cohort between the different breast cancer subtypes. Lowest median expression is seen in the basal subtype, whereas the highest is seen in the tumors without classable subtype. (C) Shows the distribution of DYX1C1 between the Elston grades in the Uppsala cohort. The expression was lowest in tumors graded 3. (D) Shows the distribution of DYX1C1 between the Elston grades in the Stockholm cohort. The expression was lowest in tumors graded 3.

When analyzing the expression of DYX1C1 mRNA using ANOVA among the different subtypes proposed by Sørlie *et al. *[1], there were significant differences (p < 0.001) between the subtypes. The subtypes were categorized according to their distinct mRNA expression profile [1]. In the Uppsala patient cohort, the lowest DYX1C1 expression was seen in the basal subtype tumors, considered to have the worst prognosis. The highest expression was found in the luminal A subtype, associated with increased survival [1] (Figure 3).

Tumors characterized as highest grade (Elston grade 3) in the Uppsala patient cohort had a significantly lower expression of DYX1C1 compared with tumors characterized as either grade 1 or 2 (p < 0.001) (Figure 3). Similar results for the subtype (p < 0.01) and Elston grading (p < 0.01) were seen in the Stockholm patient cohort (Figure 3). The analysis of DYX1C1 expression among the subtypes could not be performed for the CAHRES patient cohort as there were no microarray data available for this patient material. In addition, there was no significant correlation of DYX1C1 with the Elston grade within the CAHRES patient material (p = 0.15, data not shown).

#### Multivariate linear regression analysis

The multivariate linear regression model fitted to the DYX1C1 mRNA expression data from the Uppsala patient cohort showed that ERα, PR, and lymph node status were significant independent predictive variables of DYX1C1 mRNA expression (all p < 0.01). Elston grade status was not in itself a significant variable in the model (p = 0.50) but was as an interaction factor together with ERα (p < 0.05). Furthermore, the final model also contained the interaction factor between PR and lymph node status.

### IHC detection of DYX1C1 protein in breast cancer tumors and normal breast tissue

**Figure 4:**
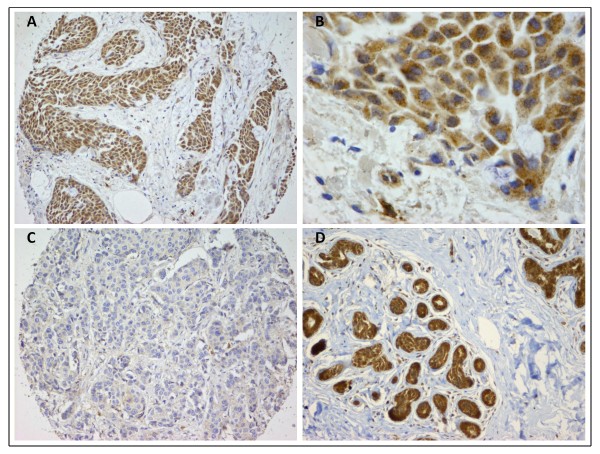
**Photomicrograph of immunohistochemical staining with antibodies against DYX1C1**. (A) Patient graded as positively expressing DYX1C1. Almost all of the cancer cells express DYX1C1, the stromal tissue is not stained. (B) Higher magnification of the same patient as in A, staining is cytoplasmic. (C) Patient graded as negative for DYX1C1 expression. (D) Tissue from healthy donor displaying normal mammary tissue, DYX1C1 expression is seen in the epithelial cells.

Tissue microarrays (TMAs) from the Uppsala breast cancer patient cohort were stained immunohistochemically using antibodies raised against DYX1C1. Each tumor was represented on the TMAs by two sections of tissue. The staining was graded according to the Allred scoring system and a score of 1 or higher was used as a cut-off to differentiate between DYX1C1-positive, and no score as negative tumors. According to this cut-off, 196 (88.7%) tumors were scored as expressing DYX1C1 and 25 (11.3%) did not express the protein (Figure 4).

#### Kaplan-Meier and Cox proportional hazard model

**Figure 5:**
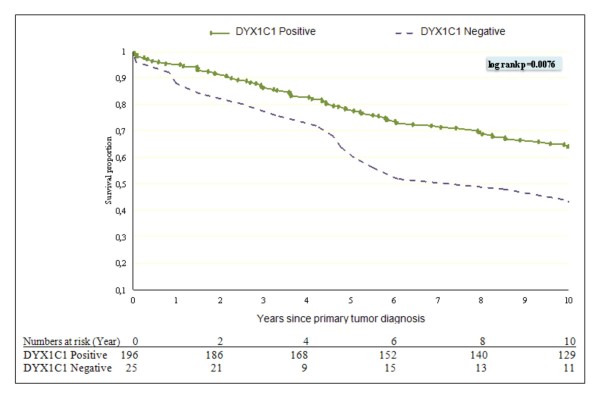
**Kaplan-Meier plot of Overall Survival for DYX1C1 expression**. Overall survival from the time of primary breast cancer diagnosis to death or censoring contrasting the DYX1C1 expression in primary tumor with a threshold greater or equal to 1 as scored by the Allred method classified as positive DYX1C1 expression with negative DYX1C1 expression. The number of patients at risk is shown.

We constructed a Kaplan-Meier univariate survival estimate stratifying for DYX1C1 expression. This showed a significant difference in overall survival between DYX1C1-positive and -negative tumors (log rank 0.0076) (Figure 5).

In the Cox proportional hazards survival model (adjusting for age at diagnosis, ER, PR, grade, and lymph node status) from primary breast cancer diagnosis to death or censoring at end of follow-up, patients with a low DYX1C1 status had a statistically significant increased risk of mortality compared with patients with high DYX1C1 status (Hazard ratio [HR] 3.44, CI 95% 1.84-6.42). However, unlike DYX1C1 mRNA data, the DYX1C1 protein expression was not associated with ERα, PR, or lymph node status of the patients, or indeed any other clinical parameter (data not shown).

To examine the normal expression of DYX1C1 in mammary tissue we stained normal tissue from 10 healthy donors obtained from breast reduction surgery. All 10 examined tissue specimens were found to strongly express DYX1C1. The expression was present in the cytoplasm of the epithelial cells, however, the surrounding stromal cells were clearly negative (Figure 4).

## Discussion

DYX1C1, a gene originally associated with dyslexia [9] and neuronal migration, is expressed in several tissues, e.g., testis, ovary and brain [9]. Two recent studies have suggested a role for DYX1C1 as a cancer biomarker [7,8]. In the present study, we report that DYX1C1 is expressed on the mRNA level in breast tumors and is associated with several clinicopathological variables such as ERα, PR, and lymph node status. We also show that the DYX1C1 protein is expressed in these tumors and is connected to the overall survival of the patients. This further points towards the potential of DYX1C1 as a breast cancer biomarker of predictive and prognostic significance.

There are several proliferative diseases of the mammary gland, both benign and malignant. The pathological mechanisms leading to malignant transformation of normal mammary epithelium are not fully understood, however mutations of tumor suppressor gene *TP53 *and amplification of *ERBB2 *are common genetic alterations. Fibroadenoma is the most common benign hyperplasia in the mammary gland, especially during adolescence. Estrogen is considered a putative agent for the development of benign breast tumors and important for growth of malignant tumors of the breast. However, females with a history of fibroadenoma do not have a higher lifetime risk of breast cancer and there is little evidence that fibroadenoma is a precancerous stage (reviewed in [23]).

Development of malignant invasive breast carcinoma is considered as a continuous process from normal epithelium, flat epithelial atypia (FEA), atypical ductal hyperplasia (ADH), ductal carcinoma *in situ *(DCIS) to invasive ductal carcinoma. Along the process, there are both loss and amplification of several genomic regions [24]. DCIS is defined as a neoplastic proliferation of epithelial cells within the duct with intact basement membrane. DCIS is generally considered as a premalignant lesion, and women with DCIS are at higher risk of developing invasive ductal carcinoma. However the precise mechanism of progression from DCIS to invasive cancer is unknown [25]. It has been shown that most expressional changes seen in invasive carcinoma are present already in DCIS [26]. An interesting example is the overexpression of CXCL12 in DCIS as well as invasive cancer, a chemokine that has been shown to increase the expression of DYX1C1 in a prostate epithelial cell line [27]. The role and expression of DYX1C1 in DCIS, premalignant and benign proliferative disorders of the mammary gland is not known and needs further study.

In this study we show by using qRT-PCR that the mean expression of DYX1C1 is 3.2 times higher in tumors that are classified as ERα-positive. We were able to replicate this association in two different independent data sets where microarray analysis of mRNA expression had been performed. Here the difference in expression was less pronounced, most likely due to the higher sensitivity of the qRT-PCR method compared with microarrays. Although several mechanisms of DYX1C1 actions have been proposed for dyslexia, the function of the gene in other diseases is still poorly understood. DYX1C1 has, however, been shown to interact with protein chaperone Hsp70, which is often up-regulated in several neoplasms and is considered a future pharmaceutical target [28]. Furthermore, when overexpressed in the neuroblastoma cell line SH-SY5Y, DYX1C1 has been shown to interact with and regulate the levels of ERα, as well as affect the migration properties of the cells [12,29]. As DYX1C1 has been shown to interact as a chaperone with ERα and also regulate its expression, higher levels of DYX1C1 protein, which could be a result of increased transcription of DYX1C1, could perhaps influence the levels of ERα and, consequently, affect the response to the anti-estrogen treatment of the patient. DYX1C1 expression is also higher in PR-positive tumors. As PR is a target gene for ERα and is considered as a marker for endocrine sensitivity, this finding is not surprising (reviewed in [30]). Both ER and PR status was independently correlated with DYX1C1 status when included into a multivariate linear regression model, further indicating that the correlation to DYX1C1 expression was a result of the receptor status independently.

Tumors classified as basal subtype, associated with the worst survival prognosis, were also the group that, on average, expressed the lowest levels of DYX1C1 mRNA. The tumors of the basal subtype lack amplification of *ERBB2 *gene (HER2) and are both ERα- and PR-negative, which could explain this association. DYX1C1 expression was on the other hand highest in the Luminal A group, which is characterized by expression of ERα, PR, low expression of proliferative genes and no HER2 amplification [24].

When correlating DYX1C1 mRNA level to the Elston grade of the tumor, the grade 3 tumors, shown to have the worst prognosis [31], had significantly lower levels of DYX1C1 compared with grades 1 and 2 combined. However, this finding was only seen in the Uppsala patient cohort and not in CAHRES. This discrepancy could be due to the enrichment of invasive tumors within CAHRES, where only 3 out of 61 patients were graded as Elston grade 1, render in inadequate power. Also, in our multivariate linear model, Elston grade was not a significant independent factor. Taken together, these results indicate that DYX1C1 mRNA is lost in high-grade aggressive tumor subtype. Our results are opposite to the published data from Chen *et al. *showing reduced DYX1C1 expression in benign tumors and normal tumor adjacent tissue compared with cancer [8]. The reason for this difference is not clear to us. It should be pointed out that we have been using different methods in detecting mRNA expression. While we were using a TaqMan probe together with qRT-PCR, Chen *et al. *used semi-quantitative PCR with designed probes. Also, a basic local alignment search tool (BLAST) analysis of the primers used by these authors revealed that their primer pairs were unable to detect one of the RefSeq isoforms of DYX1C1. It is therefore likely that they were not able to measure all the DYX1C1 mRNA.

Although patients retaining DYX1C1 expression seem to have less aggressive tumors, they do, on the other hand, have an increased tendency of lymph node metastasis which seems contradictory. As DYX1C1 has previously been shown to have a potential role in the migration of neuronal cells [29], perhaps DYX1C1 has a similar role in breast cancer cells. Reelin, another protein important in neuronal migration in the brain, was recently shown to be epigenetically silenced in breast cancer compared with normal mammary tissue [32]. Loss of Reelin protein expression was associated with decreased survival [33]. Furthermore Roundabout homolog 1 (ROBO1), also implemented in familiar dyslexia, has been identified in the progression of several cancers. ROBO1 is a membrane bound receptor that interacts with members of the SLIT family of secreted proteins important in the migration of neurons [34]. The binding of SLIT to ROBO-receptors has been shown to increase the migration of breast cancer cells and also in selection of metastasis to the brain [35]. ERα-positive tumors are considered to be less prone to metastasize, suggesting DYX1C1 as a possible marker of metastasis risk within the group of ERα-positive tumors. However, it must be noted that we were unable to replicate the association of DYX1C1 in lymph node metastasis in any of the microarray datasets.

We stained 10 healthy donors using IHC with DYX1C1 antibodies raised against DYX1C1. As DYX1C1 was seen strongly expressed in all donors it seems unlikely that the levels of DYX1C1 protein is low in normal mammary tissue. The staining was, on the contrary, usually strong. We also stained 221 tumor samples using the same antibody. This indicated that 88.7% of tumors expressed DYX1C1 protein at some level, but the expression was lost in 11.3% of the tumors. The reason for this loss is unknown to us, but perhaps it could be a regulation through the ubiquination ligase CHIP as seen in cell lines [10].

Patients with tumors classified as negative DYX1C1 protein expression by IHC had significantly higher mortality when controlling for several possible confounding factors, indicating that lost DYX1C1 expression is a poor prognostic factor. However, although DYX1C1 mRNA expression was associated with several clinicopathological variables we could not show similar associations on DYX1C1 protein level. This could be a result of a post-translational modification of DYX1C1 causing a non-linear correlation of mRNA and protein. This is not an uncommon observation and is seen with many genes (reviewed in [36]).

On the other hand, the lack of concurrence between DYX1C1 protein and mRNA results could also be a result of not choosing the appropriate cut-off for defining a positively expressing tumor. When we instead used the median of the Allred score as a cut-off for positively or negatively DYX1C1 expressing tumors we saw a correlation with the lymph node status of the patients, but not with overall survival (data not shown).

## Conclusion

Our results indicate that DYX1C1 mRNA is more highly expressed in ERα-positive or PR-positive breast cancer tumors. DYX1C1 expression is also higher in patients diagnosed with at least one lymph node metastasis and in patients with periglandular growth, suggesting that DYX1C1 could play a role in regulating ERα in the positive tumors and perhaps also in the migration of tumor cells.

The DYX1C1 protein is expressed in both normal mammary tissue and in breast cancer but is lost in some tumors. Patients who have lost DYX1C1 expression have a poor overall survival compared with patients who have retained the expression of the protein. Our data points towards DYX1C1 as an important factor in breast cancer and its expression affects breast cancer outcome. We therefore propose DYX1C1 as a possible cancer biomarker for poor survival. However, the mechanism of actions for DYX1C1 is not known and needs further investigation.

## Abbreviations

DYX1C1: Dyslexia susceptibility 1 candidate 1; IHC: Immunohistochemistry; ERα: Estrogen receptor alpha; PR: Progesterone receptor; qRT-PCR: Quantitative reverse transcriptase polymerase chain reaction; DCIS: Ductal carcinoma in situ; FEA: Flat epithelial atypia; ADH: Atypical ductal hyperplasia.

## Competing interests

The authors declare that they have no competing interests.

## Authors' contributions

GR, UH, LL, PH, JB, JH and JK designed the study. GR performed the qRT-PCR and IHC experiments. PH and JB contributed with patient materials. GR, JH and LL conducted the statistical analysis and modeling. GR drafted the manuscript and all authors revised and approved it.

## Pre-publication history

The pre-publication history for this paper can be accessed here:

http://www.biomedcentral.com/1471-2407/12/79/prepub
